# Differential Diagnosis between Takotsubo Syndrome and Acute Coronary Syndrome—A Prospective Analysis of Novel Cardiovascular Biomarkers for a More Selective Triage

**DOI:** 10.3390/jcm11112974

**Published:** 2022-05-25

**Authors:** Albert Topf, Moritz Mirna, Vera Paar, Lukas J. Motloch, Nina Bacher, Marcus Franz, Uta C. Hoppe, Daniel Kretzschmar, Michael Lichtenauer

**Affiliations:** 1 Clinic for Internal Medicine II, University Hospital Salzburg, Paracelsus University Salzburg, Müllner Hauptstraße 48, A-5020 Salzburg, Austria; m.mirna@salk.at (M.M.); v.paar@salk.at (V.P.); l.motloch@salk.at (L.J.M.); n.bacher@salk.at (N.B.); u.hoppe@salk.at (U.C.H.); m.lichtenauer@salk.at (M.L.); 2 Department of Internal Medicine I, Division of Cardiology, University Hospital Jena, 07743 Jena, Germany; marcus.franz@med.uni-jena.de (M.F.); daniel.kretzschmar@med.uni-jena.de (D.K.)

**Keywords:** Takotsubo syndrome, acute coronary syndrome, biomarkers

## Abstract

Introduction: Takotsubo syndrome (TTS) is clinically indistinguishable from an ACS. Despite the implementation of clinical scoring systems and novel biomarkers, coronary angiography currently remains necessary for differential diagnosis. Methods: 93 patients with chest pain and the suspicion of TTS were enrolled in two study centers. Fetuin-A, IGFBP-2, Galectin-3, and TNF α were determined in serum samples, collected within 24 h after the onset of symptoms. Serum levels of biomarkers were analyzed for the differential diagnostic value between TTS and ACS. Results: Compared to TTS, patients with ACS had significantly lower serum levels of Fetuin-A and IGFBP-2. The cut-off value of Fetuin-A for the identification of TTS compared to ACS was 55.74 μg/mL (sensitivity: 100.0%, specificity: 82.6%, PPV: 63.2%, NPV: 100.0%). An optimal cut-off value for IGFBP-2 for the differential diagnosis between TTS and ACS was determined as 171.77 ng/mL (sensitivity: 76.0%, specificity: 82.6%, PPV: 76.4%, NPV 72.7%). Conclusion: Fetuin-A and IGFBP-2 might facilitate the triage between TTS and ACS and could be therefore of great benefit for the guidance of treatment.

## 1. Introduction

Takotsubo syndrome (TTS) is an acute heart failure condition. Despite symptoms analogical to an acute coronary syndrome (ACS), there is no significant coronary stenosis found [1]. In three percent of all suspected ACS, TTS is responsible for the patient’s clinical presentation. A higher incidence is found among postmenopausal women [2] and emotional and physical stress factors often precede TTS. Reversible wall motion abnormalities are observed in TTS, causing either apical, midventricular, or basal hypokinesia of the left ventricle [3].

The pathophysiological background of TTS remains partly unclear. There is a large consensus that excessive epinephrine release leads to myocardial stunning [4].

Generally, TTS usually has an excellent prognosis with a convalescence of the cardiomyopathy within a few days in 96% of the affected patients [5]. In the acute phase, TTS patients can experience life-threatening complications (1–2% mortality). There is a 20% risk of cardiac decompensation, an 8.6% risk of life-threatening ventricular arrhythmias and an even small risk for left ventricular wall rupture, thrombosis, or cardiogenic shock in the acute phase [6].

The remaining clinical issue is that TTS is clinically indistinguishable from an ACS. Although established scoring systems for the prediction of TTS are available, coronary angiography currently remains necessary for differentiation between these two syndromes. Thus far, biomarkers have failed to become established in clinical routines for differential diagnosis in this regard [7].

In this study, novel cardiovascular biomarkers were selected to be investigated for their differential diagnostic purpose in TTS. Markers with diagnostic value in other cardiovascular diseases, including heart failure and acute coronary syndrome, were chosen [8,9].

**TNF α.** Among the investigated markers, tumor necrosis factor-alpha (TNF α) is the best-studied and most frequently used biomarker in clinical practice. TNF α is an inflammatory cytokine, which is synthesized in various blood, endothelial, and smooth muscle cells, as well as in cardiomyocytes [10]. TNF α is a cytokine, which is rapidly secreted in the myocardium during acute myocardial ischemia in the setting of ACS and in the development of heart failure. It is of great interest, however, that in ACS patients, the release of TNF α is not only limited to inflammatory cells of the infarction area but is also observed in healthy myocardium. TNF α has a negative inotropic effect and its level is associated with the severity of heart failure and the extent of myocardial damage [11].

**IGFBP-2.** IGFBP-2 belongs to the IGFBP family. The expression of this protein is the highest in the heart and the liver (heart > liver). It has an influence on the transport and the bioavailability of insulin-like growth factor-1 (IGF-1) actions [12]. Inhibitory effects on IGF-1 are caused by IGFBP-2 and thereby prevent the inactivation of the PI3K/Akt pathway. The inactivation of the PI3K/Akt pathway results in a proliferative and antiapoptotic effect on the myocardium. Additionally, IGFBP-2 has regulatory effects on gene expression. In the presence of oxidative stress, IGFBP leads to a parallel increase in VEGF release. Therefore, IGFBP-2 has a VEGF-mediated proliferative and antiapoptotic effect, too [13]. Despite its influence on low density lipoprotein (LDL) levels, triglycerides, and diabetes, IGFBP-2 is a less affected biomarker than IGFBP-1. According to previous studies, IGFBP-2 concentrations correlate negatively with intima–media thickness and with pulse wave velocity and thus with arterial stiffness, or rather, atherosclerosis. Therefore, IGFBP-2 identifies individuals with high cardiovascular risk, and a strong association with acute myocardial infarction was described. In heart failure, the inhibition of IGF-1 has cardioprotective effects by downregulation of the renin–angiotensin system [14].

**Fetuin-A.** Fetuin-A is a phosphorylated glycoprotein, which has the function of an antagonist of proinflammatory cytokine production [15]. Fetuin-A is mainly expressed by hepatocytes, but may also be synthesized in the kidneys and the tongue. Serving as a mediating signal for antagonizing growth factors, Fetuin-A reduces the mineralization of the skeletal matrix. By binding cationic ions, such as calcium, Fetuin-A may inhibit ectopic calcification. In previous studies, low Fetuin-A concentrations have been reported to be associated with cardiovascular death and the prognosis of patients with ACS. Decreased serum Fetuin-A levels have an influence on cardiac function by increasing cardiac fibrosis and calcification and thus promote cardiovascular disease progression [16,17].

**Galectin-3.** Galectins are divided into 3 types because of their chemical structure. It is synthesized in endothelial cells, epithelial cells, activated microglia, inflammatory cells (mainly macrophages), and various tissues, including the spleen, stomach, colon, liver, kidney, heart, uterus, and ovary [18]. Levels of Galectin-3 are associated with the risk of atherosclerosis. Galectin-3 affects the risk of atherosclerotic plaque formation and destabilization. Galectin-3 levels are increased in acute coronary syndrome. Galectin-3 concentrations could be part of the survival mechanism of the injured myocardium [19,20].

The aim of this study is to investigate the differential diagnostic value of these novel biomarkers to distinguish TTS from ACS.

## 2. Materials and Methods

**Patients and controls.** The study was approved by the local ethics committee (415-E/2230/10-2018) and was performed in accordance with the Declaration of Helsinki and Good Clinical Practice. All patients provided written informed consent prior to enrollment.

In this prospective study, 93 patients, hospitalized for chest pain and the suspicion of TTS, were enrolled in 2 study centers in Salzburg and Jena. A total of 52 TTS patients with fulfilled Mayo Clinic Diagnostic Criteria for TTS were recruited [21]. A total of 41 patients with an ACS were enrolled. ACS was diagnosed and treated in accordance with the European Society of Cardiology criteria [22].

Serum samples were collected within 24 h after the onset of symptoms. Data on clinical presentation, precipitating factors, cardiovascular risk factors, medications, and demographics were obtained as well.

**Blood samples.** The collection tubes were centrifuged within 20 min after blood collection and the obtained samples were frozen at −80 °C until further analysis was performed. Additionally, routine blood analysis was performed.

**Transthoracic echocardiography.** Transthoracic echocardiography at baseline (Philips iE 33 ultrasound system) was performed to assess left ventricular ejection fraction (LVEF). Standard echocardiographic views, including the parasternal long axis view, parasternal short axis view, and apical four chamber view, were used as previously published [23].

**Biomarker analysis.** Serum biomarker analysis was performed at baseline. Levels of IGFBP-2, Galectin-3, Fetuin-A, and TNF α were measured by using commercially available enzyme-linked immunosorbent assay (ELISA) kits (DuoSet ELISA, DY523B, R&D Systems, Minneapolis, MN, USA). In accordance with the instructions supplied by the manufacturer, ELISA assays were performed. Serum samples and standard proteins were added to the multiwell plate coated with the respective capture antibody and incubated for 2 h. Afterward, the plates were washed using washing buffer (Tween 20, Sigma Aldrich, St. Louis, MO, USA) and phosphate-buffered saline solution. Then, a biotin-labelled antibody was added to each well and incubated for an additional 2 h. After incubation, the ELISA plates were washed and a streptavidin-horseradish-peroxidase solution was added. After adding tetramethylbenzidine (TMB; Sigma Aldrich, USA), a color reaction was achieved. Optical density was measured at 450 nm on an ELISA plate reader (iMark Microplate Absorbance Reader, Bio-Rad Laboratories, Vienna, Austria). Laboratory results may be received within 4.5 h.

**Statistical analysis.** SPSS (22.0, SPSS Inc., Chicago, IL, USA) was used to perform statistical analysis. The distribution of data was assessed by the Kolmogorov–Smirnov test. As most parameters and biomarker concentrations were not normally distributed, all values were given as a median and interquartile range (IQR). Median values between groups were compared by a Mann–Whitney U test or Kruskal–Wallis test with Dunn’s post hoc test. By using Spearman’s rank correlation coefficient, the correlation was performed. ROC analysis was performed and an optimal cut-off was calculated by means of the Youden Index. Areas under the curve (AUC) were compared as described by Hanley and McNeil [24]. A *p* < 0.05 was considered to be statistically significant.

## 3. Results

**Baseline characteristics.** Baseline characteristics of patients suffering from TTS or ACS are shown in Table 1. TTS patients were non-significantly older than patients with ACS (*p* = 0.428). Female patients were almost similarly distributed between the TTS (94.2%, *n* = 49) and ACS subgroup (92.7%, *n* = 38). Left ventricular ejection fraction of TTS patients did not significantly differ from ACS patients (*p* = 0.678), and hs-troponin levels were significantly higher in ACS compared to TTS (*p* < 0.001). The apical type of TTS was the most frequent (90.4%, *n* = 47), followed by the midventricular (7.7%, *n* = 4) and the basal type (1.9%, *n* = 1). A total of 11 out of 52 patients had preceding emotional triggers. A total of 21 out of 52 patients did not have a coronary artery disease in the acute coronary angiography and the rest had nonsignificant coronary artery stenosis. In the ACS subgroup, LAD was the main culprit lesion (85.4%, *n* = 35), followed by RCX (9.8%, *n* = 4) and RCA (4.9%, *n* = 2). A total of 70.7% were diagnosed as a STEMI.

ACS patients showed a nonsignificant trend towards higher serum LDL cholesterol (*p* = 0.085) and significantly higher plasma levels of HbA1c (*p* = 0.001) than TTS patients. Regarding comorbidities, smoking, and BMI were more prevalent in patients with ACS compared to TTS patients.

**Figure 1:**
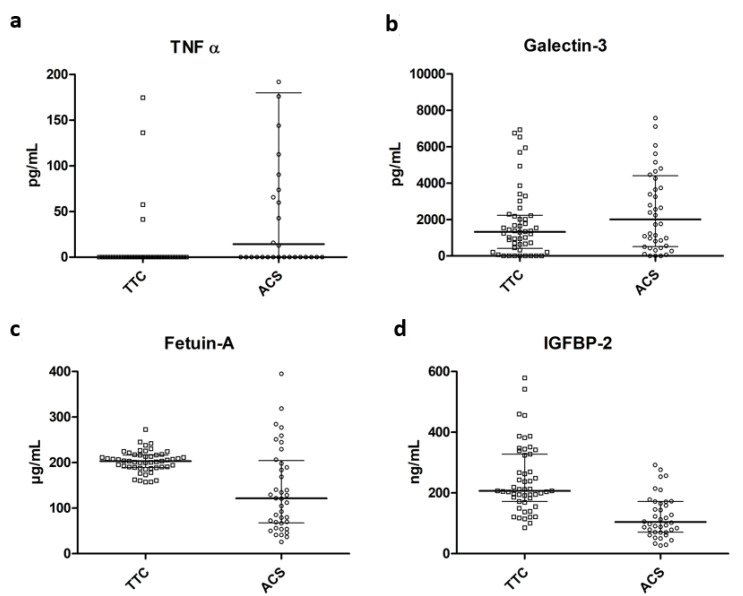
Comparison of biomarker levels between Takotsubo syndrome (TTC) and ACS patients. (**a**) TNF α. (**b**) Galectin-3. (**c**) Fetuin-A. (**d**) IGFBP-2.

**Biomarkers.** TNF α was significantly increased in patients with ACS at baseline compared to TTS patients (*p* = 0.002 see Figure 1 and Table 1) whereas IGFBP-2 and Fetuin-A were significantly elevated in TTS patients compared to the ACS subgroup (*p* ≤ 0.0001). There was no significant difference between Galectin-3 levels at admission between patients with a TTS and an ACS (*p* = 0.129, see Table 1).

**Correlations.** Correlations between biomarkers and patient characteristics are shown in Table 2. Except for IGFBP-2, no correlation of biomarkers with age was found. Only IGFBP-2 correlated with BMI and solely Galectin-3 showed a correlation with serum creatinine levels. Fetuin-A and IGFBP-2 correlated inversely with a left ventricular ejection fraction. Only IGFBP-2 showed a correlation with CRP levels and Fetuin-A, as well as IGFBP-2, and had an inverse correlation with HbA1c. Except for Galectin-3, all the other biomarkers correlated with LDL levels (see Table 2).

**Figure 2:**
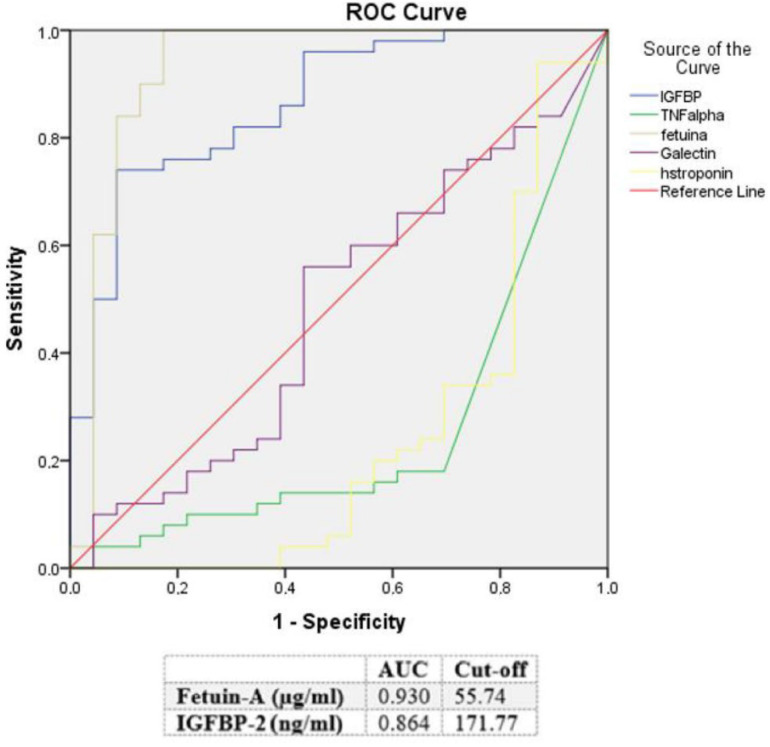
ROC curves and cut-off scores for IGFBP-2 (IGFBP), TNF α (TNF alpha), Galectin-3 (Galectin), Fetuin-A (fetuina), and high sensitive troponin (hstroponin) for prediction of ACS in the total cohort (including patients with ACS and TTS).

**ROC analysis.** Moreover, a ROC analysis was performed and AUC was calculated for IGFBP-2, TNF α, Fetuin-A, and Galectin-3 levels as differential diagnostic indicators for patients presenting with chest pain with the suspicion of TTS. In this analysis, TNF α was identified as the paramount biomarker for identification of an ACS when discriminating toward a TTS (AUC: 0.746, *p* = 0.002, see Figure 2). An optimal cut-off for diagnosis of an ACS was calculated as 6.36 pg/mL (sensitivity: 69.6%, specificity: 82.0%). Compared to TNF α, an optimal cut-off value for hs-troponin for identification of an ACS in differential diagnosis to a TTS was determined as 241.5 pg/mL (sensitivity: 82.6%, specificity: 64.0%). In contrast, Fetuin-A seemed to be the most suitable biomarker (AUC: 0.930, *p* ≤ 0.001) for the prediction of a TTS in differential diagnosis to an ACS. An optimal cut-off was 55.74 µg/mL (sensitivity: 100%, specificity: 82.6%). The second biomarker with a benefit for the identification of a TTS in patients presenting with chest pain and the suspicion of a TTS was IGFBP-2 (AUC: 0.864, *p* ≤ 0.001). The optimal cut-off value was determined as 171.77 ng/mL (sensitivity: 76.0%, specificity: 82.6%). In contrast to the other investigated biomarkers, Galectin-3 was neither significant for the prediction of an ACS, nor for the prediction of TTS. Rates for sensitivity, specificity, positive, and negative predictive values for tested biomarkers are shown in Table 3.

## 4. Discussion

### 4.1. Clinical Implementation

TTS is an acute heart failure condition with symptoms similar to an ACS. Clinical symptoms, ECG alterations, and changes in standard laboratory parameters resemble an ACS [25]. For differential diagnosis, coronary angiography has so far been necessary to distinguish TTS from ACS [26]. Biomarker analysis, combined with scoring systems, such as the InterTAK Diagnostic Score, appears to be a promising approach for a better triage [27]. Therefore, we analyzed novel biomarkers with approved diagnostic effects in cardiovascular diseases to identify TTS in patients with chest pain and to effectively determine those needing urgent coronary angiography. Especially in patients with high bleeding risk, indicators to avoid coronary angiography might be warranted. In particular, patients with neurogenic TTS due to a stroke or cerebral bleeding, or multimorbid patients with advanced renal failure, might benefit from better differential diagnosis by biomarker determination.

Routinely used cardiovascular biomarkers are not effective in differentiation between TTS and ACS. The early B-type natriuretic peptide (BNP)/troponin T (TnT) ratios (specificity: 95%, sensitivity: 52%) and BNP/Creatinkinase-MB (CK-MB) ratios (95% specificity, sensitivity 50%) have been analyzed for differential diagnosis between TTS and ACS [28]. The clinical use is limited due to the ratios’ low sensitivities. In a previous study, the analysis of circulating microRNAs (miRNAs) also showed promising results. Four miRNAs were detected to have a diagnostic value in the distinction between TTS and ACS [29]. However, despite its publication in 2014, the miRNAs measurement had not been implemented in clinical routine to differentiate TTS from ACS, most likely due to the time and costs intensive analysis.

The aforementioned study results underline the need for novel biomarkers for differential diagnosis between both disease entities. In various studies, Fetuin-A, Galectin-3, IGFBP-2, and TNF α have been investigated in cardiovascular and inflammatory diseases. We sought to determine the serum concentration of Fetuin-A, Galectin-3, IGFBP-2, and TNF α for their differential diagnostic aspects in patients with chest pain and suspicion of a TTS.

### 4.2. Interpretation of Our Results

**IGFBP-2.** IGFBP-2 levels were significantly higher in TTS patients compared to ACS patients. A ROC analysis for the identification of TTS patients in the cohort of TTS and ACS patients revealed (AUC: 0.864; *p* ≤ 0.001; cut-off value: 171.77 ng/mL with sensitivity: 76.0%, specificity: 82.6%, PPV: 76.4%, NPV 72.7%) IGFBP-2 as one of the most effective diagnostic biomarkers in this study for differential diagnosis. When considering that values of IGFBP-2 of healthy volunteers range from 300 to 500 ng/mL, lower IGFBP-2 concentrations in ACS patients compared to TTS patients reflect the higher cardiovascular risk, as previously mentioned in other studies [14].

**TNF α.** Baseline serum concentrations of TNF α in ACS patients were significantly higher compared to TTS patients (*p* = 0.002; AUC: 0.746). The cut-off value of TNF α for the identification of ACS compared to TTC was 6.36 pg/mL (sensitivity: 69.6%, specificity: 82.0%, PPV: 66.6%, NPV: 72.9%). According to the company’s information, TNF α values of healthy volunteers range from not detectable to 9.03 pg/mL. These observations are in accordance with our presumptions that TNF α is elevated by the rapid synthetization in the myocardium during acute myocardial ischemia. TNF α has a negative inotropic effect and is directly associated with the extent of myocardial damage after ischemia [11], which could also be an explanation for the inverse correlation with LV systolic function found in our study (see Table 2).

**Galectin-3.** High concentrations of Galectin-3, measured in ACS patients in our study, are in accordance with previous studies. Elevated levels of Galectin-3 are connected to atherosclerosis and to the risk of atherosclerotic plaque formation, as well as destabilization. Galectin-3 is released in acute coronary syndrome during the acute phase of acute myocardial infarction [19]. However, in our study, we found no statistically significant difference in plasma levels of Galectin-3 between the two subgroups investigated, probably due to the low number of participants enrolled.

**Fetuin-A.** Fetuin-A concentrations of TTS patients were significantly increased compared to ACS patients, indicating a high differential diagnostic value (AUC: 0.930, *p* ≤ 0.001). Fetuin-A values over 55.74 µg/mL were indicative of a TTS in the cohort of TTS and ACS patients (sensitivity: 100.0%, specificity: 82.6, PPV: 63.2%, NPV: 100.0%). When considering that values of Fetuin-A of healthy volunteers range from 303 to 671μg/mL, lower Fetuin-A levels in ACS patients compared to TTS patients reflect the higher cardiovascular risk and fibrotic processes in ACS patients, which was calculated [16].

## 5. Conclusions

Novel cardiovascular biomarkers, such as Fetuin-A, IGFBP-2, and TNF α, offer a differential diagnostic value to distinguish between TTS and ACS. Fetuin-A and IGFBP-2 are the most relevant markers with greater accuracy in a differential diagnosis as previously investigated markers in the literature and, therefore, could be beneficial for the guidance of treatment. Further large-scale studies are necessary to confirm the results of this study.

## 6. Limitations

The biggest limitation of the present study is the small study cohort, and that patients were included only in two study centers. Furthermore, patients of the subgroups were almost equally distributed regarding gender to exclude a possible bias arising from unequal distribution. As a consequence, our study results have to be validated in clinical practice, where ACS is more prevalent among male patients. Large-scale studies are necessary to confirm the results of the present study.

## Figures and Tables

**Table 1:** Baseline characteristics of patients suffering from TTC or ACS, given as median and IQR. *p* = significance between TTC and ACS patients.

	TTS		ACS		*p*
	Median	IQR	Median	IQR	
Age (years)	74.0	62.0–78.0	69.5	59.75–76.5	0.428
BMI (kg/m^2^)	24.7	21.8–29.2	28.13	24.57–33.21	0.017
EF (%)	40.0	35.0–46.0	40.5	40.0–46.5	0.678
Creatinine (µmol/L)	64.2	59.8–79.2	72.0	63.24–90.0	0.052
LDL (mg/dL)	90.0	75.0–122.0	111.54	80.77–151.92	0.085
CRP (mg/L)	0.4	0.2–0.9	0.47	0.15–1.02	0.616
HbA1c (%)	5.4	5.2–5.8	6.1	5.6–8.2	0.001
(hs) Troponin (pg/mL)	162.0	53.0–395.0	657.3	515.19–4408.07	<0.001
Galectin-3 (pg/mL)	1323.84	212.19–2232.25	2000.84	515.19–4408.07	0.129
Fetuin-A (µg/mL) 303–671 μg/mL	203.24	189.33–216.53	121.84	67.48–204.35	<0.001
IGFBP-2 (ng/mL) 300–500 ng/mL	206.93	171.82–327.58	104.23	70.53–171.65	<0.001
TNF α (pg/mL) not detectable—9.03 pg/mL	0.00	0.00–0.00	14.22	0.00–179.91	0.002
Smoking	15/52 (28.8%)		13/41 (31.7%)		
Hypertension	38/52 (73.1%)		28/41 (68.3%)		
Sex (female)	49/52 (94.2%)		38/41 (92.7%)		

**Table 2:** Bivariate correlation and point-biserial correlation analysis of baseline characteristics and biomarkers.

	Fetuin-A		IGFBP-2		TNFα		Galectin-3	
	*rs*	*p*	*rs*	*p*	*rs*	*p*	*rs*	*p*
Age (y)	0.058	0.537	0.435	<0.001	−0.156	0.108	−0.008	0.932
BMI (kg/m^2^)	−0.171	0.084	−0.311	0.001	0.126	0.230	−0.035	0.714
EF (%)	−0.446	<0.001	−0.607	<0.001	0.223	0.038	0.102	0.306
Creatinine (µmol/L)	−0.163	0.087	0.121	0.183	0.011	0.910	0.182	0.045
CRP (mg/dL)	0.024	0.802	0.436	<0.001	−0.079	0.434	0.047	0.611
LDL (mg/dL)	−0.233	0.017	−0.311	0.001	0.203	0.048	0.100	0.294
HbA1c (%)	−0.368	0.008	−0.407	0.002	0.015	0.920	0.017	0.904
Fetuin-A (µg/mL)	1.000	0.000	0.522	<0.001	−0.430	<0.001	0.099	0.289
IGFBP-2 (ng/mL)	0.522	<0.001	1.000	0.000	−0.299	0.002	−0.024	0.790
TNFα (pg/mL)	−0.430	<0.001	−0.299	0.002	1.000	0.000	0.277	0.004
Galectin-3 (pg/mL)	−0.099	0.289	−0.024	0.790	0.277	0.004	1.060	<0.001

**Table 3:** Rates for sensitivity, specificity, positive, and negative predictive values for all tested biomarkers in ACS and TTS patients.

**ACS**	**Sensitivity**	**Specificity**	**PPV**	**NPV**
TNF α	69.6%	82.0%	66.6%	72.9%
hs-troponin	82.6%	64.0%	53.5%	85.4%
**TTS**	**Sensitivity**	**Specificity**	**PPV**	**NPV**
Fetuin-A	100.0%	82.6%	63.2%	100.0%
IGFBP-2	76.0%	82.6%	76.4%	72.7%

## Data Availability

Data is available from the corresponding author upon reasonable request.
